# Healthcare Waste—A Serious Problem for Global Health

**DOI:** 10.3390/healthcare11020242

**Published:** 2023-01-13

**Authors:** Edyta Janik-Karpinska, Rachele Brancaleoni, Marcin Niemcewicz, Wiktor Wojtas, Maurizio Foco, Marcin Podogrocki, Michal Bijak

**Affiliations:** 1 Biohazard Prevention Centre, Faculty of Biology and Environmental Protection, University of Lodz, Pomorska 141/143, 90-236 Lodz, Poland; 2 Bed Management Unit, Agostino Gemelli IRCCS University Hospital Foundation, Via della Pineta Sacchetti 217, 00168 Rome, Italy; 3 European Commission, Directorate-General Migration and Home Affairs, Rue du Luxembourg 46, 1000 Brussels, Belgium; 4 Emergency Surgery Department, Fondazione Policlinico Universitario A. Gemelli IRCCS, Pineta Sacchetti 217, 00168 Rome, Italy

**Keywords:** medical waste, human health, pathogens

## Abstract

Healthcare waste (HCW) is generated in different healthcare facilities (HCFs), such as hospitals, laboratories, veterinary clinics, research centres and nursing homes. It has been assessed that the majority of medical waste does not pose a risk to humans. It is estimated that 15% of the total amount of produced HCW is hazardous and can be infectious, toxic or radioactive. Hazardous waste is a special type of waste which, if not properly treated, can pose a risk to human health and to the environment. HCW contains potentially harmful microorganisms that can be spread among healthcare personnel, hospital patients and the general public, causing serious illnesses. Healthcare personnel are the specialists especially exposed to this risk. The most common medical procedure, which pose the highest risk, is injection (i.e, intramuscular, subcutaneous, intravenous, taking blood samples). The World Health Organization (WHO) estimates that around 16 billion injections are administered worldwide each year. However, if safety precautions are not followed, and needles and syringes are not properly disposed of, the risk of sharps injuries increases among medical staff, waste handlers and waste collectors. What is more, sharps injuries increase the risk of human immunodeficiency virus (HIV), hepatitis B and C viruses (HBV/HCV), tuberculosis (TB), diphtheria, malaria, syphilis, brucellosis and other transmissions. Disposing of medical waste in a landfill without segregation and processing will result in the entry of harmful microorganisms, chemicals or pharmaceuticals into soil and groundwater, causing their contamination. Open burning or incinerator malfunctioning will result in the emission of toxic substances, such as dioxins and furans, into the air. In order to reduce the negative impact of medical waste, waste management principles should be formulated. To minimize health risks, it is also important to build awareness among health professionals and the general public through various communication and educational methods. The aim of this paper is to present a general overwiev of medical waste, its categories, the principles of its management and the risks to human health and the environment resulting from inappropriate waste management.

## 1. Introduction

**Figure 1:**
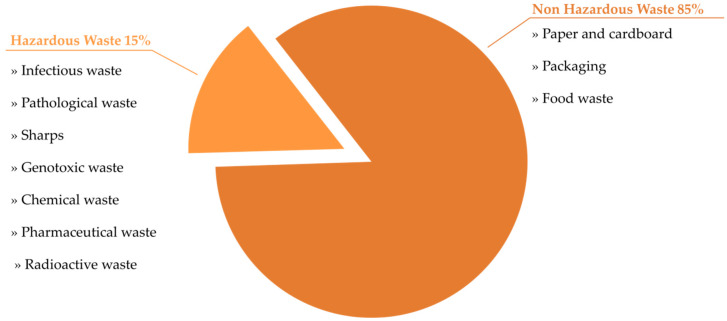
Standard waste composition in health facilities.

Healthcare facilities (HCFs) are the main healthcare waste producers. The most common term used to describe waste generated by HCFs is healthcare waste (HCW). There are several other terms such as medical waste, biomedical waste, clinical waste or health facility waste [1]. HCW is defined as all types of waste generated from HCFs, whether it is a hazardous or harmless material, and whether it is infectious or non-infectious in nature or a chemical [2]. It is estimated that HCWs constitute approx. 1–2% of total produced urban waste [3]. A total of 85% of the total amount of waste generated as a result of healthcare activities is non-hazardous. The remaining 15% are hazardous materials, which are infectious, radioactive or toxic (Figure 1). The majority of HCW generators are hospitals, medical centers, laboratories, veterinary clinics, research centers, mortuaries, blood banks and nursing homes. High-income countries produce up to almost 11 kg of hazardous waste per hospital bed per day (kg/bed/day), while in low-income countries the production rate ranges up to 6 kg. However, in low-income countries, HCW is often not segregated into hazardous and non-hazardous waste, making the actual amount of produced hazardous waste much higher [4,5].

Economic conditions are an important factor in HCW management. In many industrialized countries, institutions that generate medical waste have a legal obligation to manage this type of waste. As a result, there are appropriate structures for handling each type of waste and the amount of hazardous waste generated is constantly monitored [6]. Problems in HCW management are more prevalent in developing countries that produce several hundred tons of waste daily. Studies performed in Ethiopia revealed that 35% of healthcare institutes collect and dispose of needles, syringes and other sharp objects in a way that puts healthcare personnel and the general public at a constantly increasing risk of exposure and injury [7]. These countries typically use HCW management methods such as landfilling, recycling, incineration or storage. Although HCW landfilling without pre-treatment is prohibited, it is the most common method of HCW disposal as it is a cheap and easy method. In practice, HCW is stored in open dumps in pits mixed with municipal waste and is then incinerated [8,9]. HCW can have a long-lasting effect on human health, both for the people handling, collecting and recycling the waste, and for the general public. The environment is also suffering from fresh water and soil contamination resulting from untreated medical waste pollution or by the process of surface waste burning [6,10].

The objective of this paper is to provide a general overview of medical waste issues, including their sources and categories, waste generation, the principles of waste management and the threat to human health and the environment resulting from improper waste management.

## 2. Categories and Sources of HCW

HCW and by-products are generated as a result of diagnosis, treatment, medical intervention or the immunization of human or animals [11]. They cover a wide range of materials and different categories as summarized in Table 1 [5,8,12].

HCW is generated in various types of healthcare units, such as hospitals, medical centers, private medical practices, veterinary clinics, clinical laboratories or pharmacies [13]. Depending on the source, different types of HCW are generated, and these are summarized in Table 2 [5,14].

## 3. HCW Production Rate

The HCW production rate in countries worldwide differs and depends on many factors. These factors include waste management methods, the type of healthcare facilities, and healthcare specializations, the amount of reusable equipment available in the facility and the number of patients treated daily [15]. However, registered HCW production is lower in developing countries than in developed countries. Detailed information on the HCW production rate in different continents and selected countries are presented in Table 3.

## 4. HCW Production Rate during Pandemic

The COVID-19 pandemic has been attracting global attention since December 2019, as has the area of HCW production. The World Health Organization (WHO), Centers for Disease Control and Prevention (CDC) and local governments have announced numerous guidelines, including good hygiene practices, social distancing and quarantines, in order to reduce the spread of a new coronavirus. In addition, medical personnel and the general public have been advised to use personal protective equipment (PPE), such as surgical or medical masks, non-medical face masks (including different forms of self-made or commercial masks made of fabric, cotton or other textile materials), face shields, gloves and aprons [37]. In many countries, it is recommended to wear masks in public places. According to the press conference of the Joint Prevention and Control Mechanism of China’s Council State, the daily amount of COVID-19-related HCW in China was around 468.9 tons [38]. At the peak of the pandemic, only in Wuhan, the waste generated reached approximately 240 tons of HCW per day, almost six times more than before the pandemic [39]. In Bangladesh, in April 2020, at least 14.5 thousand tons of HCW was generated across the country due to the COVID-19 pandemic. In Dhaka, an average of 206 tons of HCW per day is generated because of the pandemic [40]. In the USA, the estimated increase in HCW generation was reported to range from 5 million tons/year before the pandemic to 2.5 million tons/month during the pandemic. The drastic increase in the number of regions, countries and people infected with SARS-CoV-2 led to global problems related to proper HCW management [41].

## 5. HCW Management

The purpose of healthcare systems is to restore health and save patients’ lives, but sometimes adverse effects on the health of healthcare personnel and communities due to unsanitary methods of disposing of HCW is observed [42]. Poorly managed waste can cause long-term and undesirable risks to public health and is a potential source of re-infection, posing a significant threat to the environment. Therefore, the management of HCW requires special attention and should be considered a high priority [43]. The management of HCW is an integral part of national healthcare systems. Safe HCW management practices reflect on HCF service quality and cover all activities related to the generation, segregation, transportation, storage, treatment and disposal of waste [44,45]. Adequate management of medical waste in HCFs depends on the waste management team, good administration and organization, careful planning, legal frameworks, adequate funding and the full participation of trained personnel in this process [46]. Healthcare facilities managers are responsible for introducing and ensuring an appropriate waste management system, as well as supervising the compliance with appropriate procedures of all medical staff. Therefore, appropriate education and training systems must be available to all personnel responsible and engaged in both segregation and waste collection processes [47,48,49]. In line with WHO guidelines, waste segregation practices should be standardized across the country and included in national regulations for HCW management [5]. The key to the effective management of HCW is the segregation process at the point of waste generation. Segregation means the separation of various types of waste into different color-coded containers with liners at places where they are generated as a first step in HCW management [50,51]. According to WHO recommendations concerning segregation and collection, a general waste container should be black. Sharp, infectious and pathological waste containers should be marked yellow. Chemical and pharmaceutical waste container should have a brown color. It is also recommended that almost all waste categories should be collected at least once per day, or when three-quarters of the container is filled. The exceptions to this are pharmaceutical, chemical and radioactive waste, which can be collected on demand [52,53].

After segregation, waste is collected and transported outside the hospital or healthcare facility. The transportation of HCW is usually performed using dedicated trolleys and containers. The trolleys have to be cleaned and disinfected daily. Hazardous and non-hazardous waste has to always be transported separately [54]. The waste should be stored in designated rooms and appropriate safety and security measures should be taken. In general, non-hazardous, infectious and sharp, pathological, pharmaceutical, chemical and radiological waste should be stored separately in different places with different characteristics depending on the waste stored [53].

## 6. HCW Management during COVID-19 Pandemic

Since March 2020, the whole world has been focusing on the COVID-19 pandemic. It has been considered whether the spread of COVID-19 could also increase as a result of inadequate waste management. Performed studies indicated that the SARS-CoV-2 survival rate on different surface varied from 4 h on copper to up to 3 days on plastic and stainless steel [55]. The increase in waste generation during the pandemic, as well as the disposal of infected disposable masks and other PPE, has burdened waste management systems [56,57,58]. Therefore, ensuring the efficient, timely and harmless management of COVID-19 medical waste has also become a significant part of pandemic controlling [59]. In addition to introduced standards, such as proper identification, collection, segregation, storage, transport, processing and disposal, aspects such as disinfection, personnel protection and training have become part of effective HCW management [57]. It has been shown that fomites may not be as critical to the transmission of SARS-CoV-2 as initially suspected [60]. At this moment, there is no significant differences between overall COVID-19 HCW management and general pre-pandemic medical waste management [38].

## 7. Risk Related to HCW

HCW is potentially dangerous and a pollutant [43]. Everyone close to hazardous medical waste is potentially at risk, including those working in healthcare facilities, those handling medical waste or those exposed through careless actions. The main risk groups are physicians, nurses, healthcare support staff, patients, HCF visitors and support services workers, such as laundry workers, waste management and transportation staff and waste-disposal facility employees [61]. Globally, more than two million medical personnel are exposed to pathogens as a result of their daily work routines [1]. In conclusion, HCW poses a serious threat to human health and life especially in low- and middle-income countries. Globally, it is estimated that at least 5.2 million people worldwide die each year, including 4 million children, due to illnesses caused by unmanaged medical waste [40].

### 7.1. Infectious Waste and Sharps

Infectious waste is a variety of hazardous waste which, due to its pathogenic nature, pose a threat to human health. It should always be assumed that infectious waste may contain various pathogenic microorganisms [62]. HCW can transmit more than 30 dangerous blood-borne pathogens [1]. Pathogens in infectious waste that is not properly managed can enter the human body through damaged skin (rubbing, puncturing or cutting the skin), inhalation, mucous membranes or by ingestion [5]. Performed research indicates the presence of various pathogens in medical waste, as well as the possibility of their transmission routes. Therefore, it can be concluded that this type of waste poses a great potential risk to human health [63,64,65].

The greatest risk of transmission of blood-borne pathogens is caused by needle stick and sharp injuries (NSSIs) [66]. It is estimated that 600,000 to 800,000 needle stick injuries and other percutaneous injuries are reported annually in the U.S.A. In addition, around 100,000 NSSIs occur in the UK each year [67]. It has been estimated that up to 30% of hepatitis B, 1–3% of hepatitis C and 0.3% of HIV cases were caused by inappropriate HCW handling [68]. HBV is more contagious than other blood-borne viral pathogens and is approximately 100 times more contagious than HIV. Consequently, HBV poses the greatest occupational risk to non-immune healthcare personnel [69]. In addition, medical waste handlers are the group more vulnerable to HBV infection than other healthcare personnel, non-medical waste handlers or the general population [70,71,72]. The performed study showed that the prevalence of HBV and HCV was significantly higher in medical waste compared to non-clinical waste handlers. The authors clearly pointed out the reason for this situation. Poor waste management systems contributed to higher acute injuries incidences and splashes of blood and body fluids [73]. A. total of 70% of the world’s HIV-infected population comes from Sub-Saharan Africa, but only 4% of global occupational cases of HIV infection are reported from this region [67]. It is estimated that up to 5% of all HIV infections in Africa are due to unsafe injection administration, including exposure to sharps injuries during unsafe medical waste handling [74]. A study conducted in China showed low risk awareness among nurses concerning the risk of HIV infection and a lack of compliance with standard precautions in daily work [75]. Over 20 other infections can also be transmitted by NSSIs, including syphilis, herpes and malaria. While most NSSIs appear in developing countries, NSSIs are still reported in developed countries despite preventive measures taken, such as standard operating protocols and real-time injury-monitoring systems [35,76,77,78]. These injuries not only increase the possibility of negative health consequences, but also lead to mental stress, fear, tension and anxiety among healthcare personnel [79]. The implementation of safety protocols and compulsory training programs for healthcare professionals can reduce the prevalence of NSSIs and associated infections [80,81].

### 7.2. Chemical and Pharmaceutical Waste

Many chemicals and pharmaceuticals used in healthcare systems can be hazardous. They are usually found in small amounts in medical waste, while larger amounts can be found when unwanted or expired chemicals and pharmaceuticals are directed for disposal [5]. Chemical waste negatively affects human health and, in most cases, causes intoxication as a primary result of contact with them. Poisoning from the absorption of a chemical or pharmaceutical substance via the mucous membranes, the skin, inhalation or ingestion is the secondary result. Contact with corrosive, flammable or reactive chemicals (formaldehyde and other volatile substances) may cause injuries to the eyes, skin or mucous membranes of the respiratory tract and should be considered thirdly [82]. Pharmaceuticals enter the environment as a result of the improper handling of unused or expired pharmaceuticals, mainly disposed of into sewage systems. Pharmaceuticals have been reported in various places, such as groundwater, surface water and soil. The main groups of pharmaceuticals detected in environmental samples are antibiotics, hormones, non-steroidal anti-inflammatory drugs, beta blockers, lipid regulators and anti-depressant drugs [83,84]. The long-term presence of pharmaceuticals in the environment causes acute and chronic damage, behavioral changes, reproductive disorders and the inhibition of cell proliferation in animals [85,86]. The negative impact of pharmaceuticals on the environment is also evidenced by the development of antibiotic resistance in some bacterial strains, resulting in an accumulation of antibiotics in the environment. Therefore, it is essential to decontaminate chemical and pharmaceutical waste before placing them in landfills, as improper disposal will cause contact between environmental bacteria and antibiotics, which can lead to the evolution of antibiotic-resistant mechanisms among them [83,87,88].

### 7.3. Genotoxic Waste

The main routes of exposure to genotoxic waste are inhalation and skin absorption. However, ingestion and accidental injection or other sharps injuries are also possible. Exposure may also occur through contact with the patient’s body fluids and secretions (such as vomit, urine and feces) while undergoing chemotherapy [89,90]. Cytotoxic drugs or anticancer drugs are classified as dangerous medicaments. Acute exposure usually causes temporary symptoms, such as dizziness, headache, nausea and malaise. What is more, cytotoxic drugs possess strong irritating properties, and direct contact will lead to the appearance of local symptoms, such as rash, dermatitis, irritation of the skin, mucous membrane ulceration and irritation of the throat or eyes [91]. The side effects from prolonged or repeated exposure to cytotoxic drugs are significant and serious. An increased incidence of spontaneous abortions during pregnancy and malformations have been observed among children of females with a history of occupational exposure to anticancer medicaments [92]. Cytotoxic drugs are also not neutral to the environment, especially the aquatic environment [93]. Some cytotoxic drugs are not fully metabolized and are poorly biodegradable. They can also be resistant to conventional biological and chemical processes used in wastewater treatments and can challenge water-decontamination technology. While aquatic cytotoxic drug concentrations may stay below detection limits, they can reach alarming levels in fauna and flora through bioaccumulation and biomagnification processes. Therefore, their effect should be carefully investigated as unexpected delayed effects can be present in offspring [94]. Kovacs et al. demonstrated that long-term exposure of zebrafish to anticancer drugs impaired their DNA integrity and induced massive whole-transcriptome changes, which might affect entire zebrafish populations [95].

### 7.4. Radioactive Waste

The disease caused by radioactive waste depends on the type and extent of exposure. This can include headache, dizziness and vomiting, as well as much more serious problems. Radioactive waste is genotoxic and, if the radiation dose is high enough, it can also affect the genetic material. Inadequate handling of radiation diagnostic instruments can cause much more serious injuries, including tissue destruction, which in some cases requires the amputation of body parts. Extreme cases can be even fatal [5,96].

## 8. HCW Treatment and Safety Issues

The most common types of HCW treatments are steam-based treatments (autoclaving, microwave and frictional heat treatments), which are used to disinfect/sterilize highly infectious and sharp waste by subjecting them to moist heat and steam. Steam sterilization is used for sterilization instruments and for sharp and hazardous waste treatments. To reduce the volume of waste, steam sterilization can be combined with mechanical processes, such as mixing, grinding and shredding [53]. Incineration, the process of waste destruction by burning, removes hazardous materials, reduces their mass and volume and converts them into ashes. An incinerator that is not properly designed or operated, or is poorly maintained, emits toxic substances into the environment. If incinerators operate at low temperatures, they generate emissions containing dioxins and furans, which may cause health problems as they are carcinogenic [97]. Incinerators operating at 850–1100 °C and containing special gas-cleaning equipment can comply with international emission dioxin and furan standards. Dioxin-control technologies use activated carbon (AC) adsorption. Before flue gas flows into the dust-collection equipment, AC is injected to adsorb the dioxin and then is blocked by a bag filter [61]. The next method used is a chemical treatment process. It mostly relies on using disinfectants, ozone treatment and alkaline hydrolysis. Composting and vermicomposting (which uses earthworms to consume and recycle the organic waste) are successfully used to break down hospital kitchen waste, as well as other digestible organic and placental waste. Another example of a biological process is the natural decomposition of pathological waste through its burial. Non-hazardous waste should be recycled and regularly collected by the municipalities or transported by the facility to public landfills [53]. Inadequate HCW treatment can be dangerous for health. Incinerator control results in the release of small particulates that affect the functioning of the respiratory and cardiovascular systems. Volatile metals, such as mercury, lead, arsenic and cadmium, will damage the immune and neurological systems, as well as the kidneys, brain and lungs. The incineration of high-metal-content materials leads to the spread of toxic metals in the environment [98,99]. Various studies have shown adverse health effects in populations in the vicinity of incinerators, including cancer and reproductive dysfunction [100,101,102]. Ashes, as a result of the incineration of hazardous medical waste, are also hazardous. Bottom ash analyses of incinerated medical waste carried out in Tanzania indicate the hazardous nature of ash resulting from the presence of large amounts of heavy metals (iron, cadmium, lead, copper and manganese) [103]. Burying medical waste and depositing them in landfills is also dangerous. Medical waste is almost always contaminated with pathogens, and leaching toxic heavy metals and chemicals from solid medical waste into the soil occurs in poorly designed dump sites and landfills. The leachate can penetrate the soil and contaminate crops, surface and groundwater resources, posing a risk to human health by consuming water. To control the safety of these methods, hydro-geological conditions must be considered. Landfills should have restricted access, control scavenging, use a soil cover regularly, manage waste discharge, and control surface water and drainage [65,104]. An interesting solution is the possibility of thermal energy, fuel, and electric-power production from medical waste, and some studies concerning this issue have been conducted. One study showed that waste-disposable syringes treated with pyrolysis at 400–550 °C were used to produce liquid fuel. The produced pyrolysis oil had physical properties similar to that of a diesel or petrol mixture [105]. Fang et al. [106] showed that the pyrolysis of mixed medical waste, such as plastic, cotton and glass, at 500 °C can produce liquid fuel (pyrolysis oil). It can be refined by fractional condensation. In a different study, biogas from recycled medical cotton waste as a source of biogas recovery, using thermophilic bio-digestion conditions, was produced. It improved biogas yield by 92% [107]. These studies bring hope that in the future it will be possible to use medical waste to produce energy or fuel on a large scale.

## 9. Conclusions

Medical waste amounts have increased dramatically over the last 30 years, and health facilities around the world are producing more waste than ever before. The amount of HCW generation is rising with the increase in the world’s population, medical facilities’ multitude and with the widespread propensity to use disposable medical equipment. Due to the use of advanced technological practices and safety considerations, single-use equipment causes more waste generation [108]. Further problems include a lack of health risk awareness associated with HCW, insufficient training in proper waste management, inadequate human resources and the low priority given to this matter [8]. Studies in developing countries have shown evidence that medical waste is mixed and collectively combined with municipal waste or burned in the open air [8,96]. Such activities pose risks to public health and the environment. Medical waste can contain potentially harmful microorganisms that can infect healthcare professionals, patients and the general public. Potential risks include drug-resistant microorganisms that spread from HCFs into the environment. Another risk is the release of toxic compounds into the environment, such as heavy metals, dioxins and furans [5,109]. In order to reduce the risk associated with medical waste, it is necessary to focus on a few key aspects. Improved policies and procedures should be developed and implemented for the proper use of single-use or reusable items and the identification of recycling options. Activities may also include working with providers to make products available in materials that are more easily degraded, or that can be reused for secondary purposes. There are items that are not hazardous (such as clean packaging) and can be removed without unnecessary treatment prior to the final disposal. Another option is to minimize the impact by adjusting purchasing strategy and inventory control. This solution can also be implemented through the use of physical (steam treatment) instead of chemical disinfection, waste minimization by using less materials and finally by checking the expiration date of the products upon delivery and refuse to accept items with a short expiration date [5,110]. Major challenges related to the risk of HCW are misconceptions and a lack of education and awareness regarding which type of waste is hazardous and which is not. In particular, educating healthcare professionals on the proper segregation and disposal of different waste types would be very beneficial to waste reduction and proper infection control [111,112]. In summary, the risks of medical waste can be significantly reduced by implementing appropriate measures. This would result in fewer illnesses and accidental sharps injuries, but also less environmental pollution.

## Figures and Tables

**Table 1:** Categories of healthcare waste.

Waste Categories	Descriptions and Examples
Hazardous HCW	
Sharps waste	Used or unused sharps, e.g., intravenous, hypodermic or other needles, syringes with attached needles, auto-disable syringes, infusion sets, scalpels, knives, blades, pipettes, broken glass and vials
Infectious waste	Waste suspected of containing pathogens and presenting a risk of disease transmission, e.g., laboratory culture and microbiological stocks, waste contaminated with blood and other body fluids, waste including excreta and other materials that have been in contact with infected patients with highly infectious diseases in isolated wards
Pathological waste	Human and animal organs, tissues or fluids, body parts, fetuses, placentas, unused blood products, contaminated animal carcasses
Pharmaceutical waste	Expired pharmaceuticals, unused, contaminated vaccines and drugs, items contaminated by or containing pharmaceuticals
Cytotoxic waste	Waste containing substances with genotoxic properties (mutagenic, carcinogenic or teratogenic substances), e.g., cytotoxic drugs used in cancer therapy and their metabolites
Chemical waste	Waste containing chemicals, e.g., expired or unused disinfectants or laboratory reagents, unused, solvents, waste with high heavy metal content, such as batteries, blood pressure gauges, mercury-containing equipment and devices (e.g., old thermometers)
Radioactive waste	Waste containing radioactives, e.g., unused radiotherapy liquids, radioactive diagnostic material, contaminated packages, absorbent paper or glassware, urine and excreta from patients tested or treated with radionuclides, radioactive sealed sources
Non-hazardous or general HCW	Waste, which does not pose any particular biological, chemical, physical or radioactive hazard

**Table 2:** Sources and examples of HCW.

Source	Sharp Medical Instruments	Infectious Waste	Medications, Chemicals and Potentially Toxic Waste	Non-Hazardous Waste
Medical department	Intravenous needles, hypodermic needles, broken ampules and vials	Bandages, dressings, gauze, gloves and masks contaminated with blood or body fluids	Broken thermometers and blood pressure gauges; split medications and disinfectants	Empty bottles, non-blood intravenous tubing, non-blood diapers and bags, packaging, flowers, paper, food scraps
Operating room	Needles, blades, scalpels, surgical power tools	Masks, gowns, gloves, gauze and medical equipment contaminated with blood and body fluids; organs and tissues	Anesthetic gases, disinfectant products	Package, uncontaminated medical clothing and medical personal protective equipment
Veterinary clinics	Needles, broken glass and vials, pipettes, Petri dishes, slides and cover slips, needles sets, blades, scalpels, syringes	Dressings, bandages, masks, gloves, sample tubes and containers contaminated with blood and body fluids; infected animal carcasses	Anesthetic gases, disinfectants, broken blood pressure gauges and thermometers, split medicaments and vaccines, dental amalgam fillings, vaccines; contaminated urine and excreta	Package, containers, paper, uncontaminated masks, gloves, hats, shoe covers and gowns, empty bottles, empty bottles, non-blood intravenous tubing, non-blood diapers and bags, paper, animal food scraps
Laboratory	Broken glass, vials, pipettes and slides	Blood and body fluids, microbiological cultures, sample tubes and containers contaminated with blood and body fluids; infected animal carcasses	Broken thermometers, chemicals, such as methanol, fixatives, formalin, toluene, xylene, medications, disinfectants	Package, containers, paper, uncontaminated masks, gloves, hats, shoe covers and gowns, empty bottles
Oncology department	Syringes and needles		Bulk chemotherapy drugs; gloves and materials contaminated with cytotoxic drugs; contaminated urine and excreta	Package, paper
Pharmacy store			Expired and split medicaments and their components; gloves	Package, paper, empty containers
Vaccination proccess	Syringes and needles		Bulk and split vaccine, vials and gloves	Package, paper, empty containers
Doctors’ offices	Syringes and needles, broken vials	Dressings, gauze, masks, gloves, sample tubes and containers, contaminated with blood and body fluids	Broken thermometers and blood pressure gauges; expired drugs and spent disinfectants	Package, paper, empty containers, newspapers, uncontaminated medical personal protective equipment
Dental clinics	Syringes and needles, broken vials	Dressings, gauze, masks, gloves, sample tubes and containers, contaminated with blood and body fluids	Dental amalgam fillings, disinfectants	Package, paper, empty containers, newspapers, uncontaminated medical personal protective equipment
Patients’ home	Insulin injection needles	Dressings and gauze contaminated with blood and body fluids	Broken thermometers and blood pressure gauges	Household waste

**Table 3:** Example of HCW production rate in various countries worldwide.

Continent	Countries	HCW Generation (kg/bed/day)	Reference
North America	USA	8.4–10.7	[16,17,18]
	Canada	8.2	[16,19]
Europe	Spain	3.5–4.4	[17,18,20]
	Norway	3.9	[18,20]
	Greece	0.3–3.6	[18,21,22]
	France	2.7–3.3	[17,18,23]
Asia	Kazakhstan	5.34–5.4	[8,18,24]
	China	0.6–4.03	[18,25,26]
	Jordan	2.5–6.10	[17,18,27]
	India	0.8–2.31	[18,23,28]
South America	Argentina	2.7–3.0	[18,23]
	Brazil	2.94–3.3	[17,18,29]
	Ecuador	2.09–2.1	[18,30]
Africa	Ethiopia	1.1–1.8	[18,31,32]
	Egypt	0.7–1.7	[18,19,33]
	Sudan	0.38–0.9	[18,34,35]
	Morocco	0.4–0.7	[18,36]

## Data Availability

Not applicable.

## References

[1] Yazie T.D., Tebeje M.G., Chufa K.A. (2019). Healthcare Waste Management Current Status and Potential Challenges in Ethiopia: A Systematic Review. BMC Res. Notes.

[2] Hasan M.M., Rahman M.H. (2018). Assessment of Healthcare Waste Management Paradigms and Its Suitable Treatment Alternative: A Case Study. J. Environ. Public Health.

[3] Dehghani M.H., Ahrami H.D., Nabizadeh R., Heidarinejad Z., Zarei A. (2019). Medical Waste Generation and Management in Medical Clinics in South of Iran. MethodsX.

[4] Taslimi M., Batta R., Kwon C. (2020). Medical Waste Collection Considering Transportation and Storage Risk. Comput. Oper. Res..

[5] Chartier Y., World Health Organization (2014). Safe Management of Wastes from Health-Care Activities.

[6] Kwikiriza S., Stewart A.G., Mutahunga B., Dobson A.E., Wilkinson E. (2019). A Whole Systems Approach to Hospital Waste Management in Rural Uganda. Front. Public Health.

[7] Meleko A., Tesfaye T., Henok A. (2018). Assessment of Healthcare Waste Generation Rate and Its Management System in Health Centers of Bench Maji Zone. Ethiop. J. Health Sci..

[8] Khan B.A., Cheng L., Khan A.A., Ahmed H. (2019). Healthcare Waste Management in Asian Developing Countries: A Mini Review. Waste Manag. Res..

[9] Ciplak N., Kaskun S. (2015). Healthcare Waste Management Practice in the West Black Sea Region, Turkey: A Comparative Analysis with the Developed and Developing Countries. J. Air Waste Manag. Assoc..

[10] Santos E.d.S., Gonçalves K.M.d.S., Mol M.P.G. (2018). Healthcare Waste Management in a Brazilian University Public Hospital. Waste Manag. Res..

[11] Kalogiannidou K., Nikolakopoulou E., Komilis D. (2018). Generation and Composition of Waste from Medical Histopathology Laboratories. Waste Manag..

[12] Xin Y. (2015). Comparison of Hospital Medical Waste Generation Rate Based on diagnosis-Related Groups. J. Clean. Prod..

[13] Khobragade D. (2019). Health Care Waste: Avoiding Hazards to Living and Non Living Environment by Efficient Management. Fortune J. Health Sci..

[14] Aljabre S.H.M. (2002). Hospital Generated Waste: A Plan for Its Proper Management. J. Fam. Community Med..

[15] Bokhoree C., Beeharry Y., Makoondlall-Chadee T., Doobah T., Soomary N. (2014). Assessment of Environmental and Health Risks Associated with the Management of Medical Waste in Mauritius. APCBEE Procedia.

[16] Sepetis A., Zaza P.N., Rizos F., Bagos P.G. (2022). Identifying and Predicting Healthcare Waste Management Costs for an Optimal Sustainable Management System: Evidence from the Greek Public Sector. Int. J. Environ. Res. Public Health.

[17] Windfeld E.S., Brooks M.S.-L. (2015). Medical Waste Management—A Review. J. Environ. Manag..

[18] Singh N., Ogunseitan O.A., Tang Y. (2022). Medical Waste: Current Challenges and Future Opportunities for Sustainable Management. Crit. Rev. Environ. Sci. Technol..

[19] Minoglou M., Gerassimidou S., Komilis D. (2017). Healthcare Waste Generation Worldwide and Its Dependence on Socio-Economic and Environmental Factors. Sustainability.

[20] Bdour A., Altrabsheh B., Hadadin N., Al-Shareif M. (2007). Assessment of Medical Wastes Management Practice: A Case Study of the Northern Part of Jordan. Waste Manag..

[21] Komilis D., Fouki A., Papadopoulos D. (2012). Hazardous Medical Waste Generation Rates of Different Categories of Health-Care Facilities. Waste Manag..

[22] Zamparas M., Kapsalis V.C., Kyriakopoulos G.L., Aravossis K.G., Kanteraki A.E., Vantarakis A., Kalavrouziotis I.K. (2019). Medical Waste Management and Environmental Assessment in the Rio University Hospital, Western Greece. Sustain. Chem. Pharm..

[23] Rabeie O.L., Miranzadeh M.B., Fallah S.H., Dehqan S., Moulana Z., Amouei A., Mohammadi A.A., Asgharnia H.A., Babaie M. (2012). Determination of Hospital Waste Composition and Management in Amol City, Iran. Health Scope.

[24] Gusca J., Kalnins S.N., Blumberga D., Bozhko L., Khabdullina Z., Khabdullin A. (2015). Assessment Method of Health Care Waste Generation in Latvia and Kazakhstan. Energy Procedia.

[25] Gai R., Kuroiwa C., Xu L., Wang X., Zhang Y., Li H., Zhou C., He J., Tang W. (2009). Hospital Medical Waste Management in Shandong Province, China. Waste Manag. Res..

[26] Zhang H.-J., Zhang Y.-H., Wang Y., Yang Y.-H., Zhang J., Wang Y.-L., Wang J.-L. (2013). Investigation of Medical Waste Management in Gansu Province, China. Waste Manag. Res..

[27] Eker H.H., Bilgili M.S. (2011). Statistical Analysis of Waste Generation in Healthcare Services: A Case Study. Waste Manag. Res..

[28] Patil G.V., Pokhrel K. (2005). Biomedical Solid Waste Management in an Indian Hospital: A Case Study. Waste Manag..

[29] Da Silva C.E., Hoppe A.E., Ravanello M.M., Mello N. (2005). Medical Wastes Management in the South of Brazil. Waste Manag..

[30] Diaz L.F., Eggerth L.L., Enkhtsetseg S. (2001). Anejo de Residuos de Establecimientos de Salud en Guayaquil, Ecuador.

[31] Tesfahun E., Kumie A., Beyene A. (2016). Developing Models for the Prediction of Hospital Healthcare Waste Generation Rate. Waste Manag. Res..

[32] Wassie B., Gintamo B., Mekuria Z.N., Gizaw Z. (2022). Healthcare Waste Management Practices and Associated Factors in Private Clinics in Addis Ababa, Ethiopia. Environ. Health Insights.

[33] Shouman E., Al Bazedi G., Sorour M.H., Abulnour A.G. (2013). Management of Hazardous Medical Waste Treatment in Egypt. World Appl. Sci. J..

[34] Saad S.A.G. (2013). Management of Hospitals Solid Waste in Khartoum State. Environ. Monit. Assess..

[35] Hassan A.A., Tudor T., Vaccari M. (2018). Healthcare Waste Management: A Case Study from Sudan. Environments.

[36] Mbarki A., Kabbachi B., Ezaidi A., Benssaou M. (2013). Medical Waste Management: A Case Study of the Souss-Massa-Draa Region, Morocco. J. Environ. Prot..

[37] Sangkham S. (2020). Face Mask and Medical Waste Disposal During the Novel COVID-19 Pandemic in Asia. Case Stud. Chem. Environ. Eng..

[38] Peng J., Wu X., Wang R., Li C., Zhang Q., Wei D. (2020). Medical Waste Management Practice During the 2019–2020 Novel Coronavirus Pandemic: Experience in a General Hospital. Am. J. Infect. Control.

[39] Singh N., Tang Y., Zhang Z., Zheng C. (2020). COVID-19 Waste Management: Effective and Successful Measures in Wuhan, China. Resour. Conserv. Recycl..

[40] Rahman M.M., Bodrud-Doza M., Griffiths M.D., Mamun M.A. (2020). Biomedical Waste Amid COVID-19: Perspectives from Bangladesh. Lancet. Glob. Health.

[41] Ilyas S., Srivastava R.R., Kim H. (2020). Disinfection Technology and Strategies for COVID-19 Hospital and Bio-Medical Waste Management. Sci. Total Environ..

[42] Arab M., Rouhollah Askari B., Tajvar M., Pourreza A., Omrani G., Mahmoudi M. (2008). Report: The Assessment of Hospital Waste Management: A Case Study in Tehran. Waste Manag. Res..

[43] Wafula S.T., Musiime J., Oporia F. (2019). Health Care Waste Management among Health Workers and Associated Factors in Primary Health Care Facilities in Kampala City, Uganda: A Cross-Sectional Study. BMC Public Health.

[44] Sahiledengle B. (2019). Self-Reported Healthcare Waste Segregation Practice and Its Correlate among Healthcare Workers in Hospitals of Southeast Ethiopia. BMC Health Serv. Res..

[45] Tsakona M., Anagnostopoulou E., Gidarakos E. (2007). Hospital Waste Management and Toxicity Evaluation: A Case Study. Waste Manag..

[46] Awodele O., Adewoye A.A., Oparah A.C. (2016). Assessment of Medical Waste Management in Seven Hospitals in Lagos, Nigeria. BMC Public Health.

[47] Anozie O.B., Lawani L.O., Eze J.N., Mamah E.J., Onoh R.C., Ogah E.O., Umezurike D.A., Anozie R.O. (2017). Knowledge, Attitude and Practice of Healthcare Managers to Medical Waste Management and Occupational Safety Practices: Findings from Southeast Nigeria. J. Clin. Diagn. Res. JCDR.

[48] Ozder A., Teker B., Eker H.H., Altındis S., Kocaakman M., Karabay O. (2013). Medical Waste Management Training for Healthcare Managers—A Necessity?. J. Environ. Health Sci. Eng..

[49] Parida A., Capoor M.R., Bhowmik K.T. (2019). Knowledge, Attitude, and Practices of Bio-Medical Waste Management Rules, 2016; Bio-Medical Waste Management (Amendment) Rules, 2018; and Solid Waste Rules, 2016, among Health-Care Workers in a Tertiary Care Setup. J. Lab. Physicians.

[50] Akulume M., Kiwanuka S.N. (2016). Health Care Waste Segregation Behavior among Health Workers in Uganda: An Application of the Theory of Planned Behavior. J. Environ. Public Health.

[51] Datta P., Mohi G.K., Chander J. (2018). Biomedical Waste Management in India: Critical Appraisal. J. Lab. Physicians.

[52] Pandey A., Ahuja S., Madan M., Asthana A.K. (2016). Bio-Medical Waste Managment in a Tertiary Care Hospital: An Overview. J. Clin. Diagn. Res. JCDR.

[53] World Health Organization (2017). Safe Management of Wastes from Health-Care Activities: A Summary.

[54] Singh H., Rehman R., Bumb S. (2014). Management of Biomedical Waste: A Review. Int. J. Dent. Med. Res..

[55] Nghiem L.D., Morgan B., Donner E., Short M.D. (2020). The COVID-19 Pandemic: Considerations for the Waste and Wastewater Services Sector. Case Stud. Chem. Environ. Eng..

[56] Mol M.P.G., Caldas S. (2020). Can the Human Coronavirus Epidemic Also Spread through Solid Waste?. Waste Manag. Res..

[57] Sharma H.B., Vanapalli K.R., Cheela V.R.S., Ranjan V.P., Jaglan A.K., Dubey B., Goel S., Bhattacharya J. (2020). Challenges, Opportunities, and Innovations for Effective Solid Waste Management During and Post COVID-19 Pandemic. Resour. Conserv. Recycl..

[58] Yang L., Yu X., Wu X., Wang J., Yan X., Jiang S., Chen Z. (2021). Emergency Response to the Explosive Growth of Health Care Wastes During COVID-19 Pandemic in Wuhan, China. Resour. Conserv. Recycl..

[59] Sarkodie S.A., Owusu P.A. (2020). Impact of COVID-19 Pandemic on Waste Management. Environ. Dev. Sustain..

[60] Meister T.L., Dreismeier M., Blanco E.V., Brüggemann Y., Heinen N., Kampf G., Todt D., Nguyen H.P., Steinmann J., Schmidt W.E. (2022). Low Risk of Severe Acute Respiratory Syndrome Coronavirus 2 Transmission by Fomites: A Clinical Observational Study in Highly Infectious Coronavirus Disease 2019 Patients. J. Infect. Dis..

[61] Padmanabhan K.K., Barik D. (2019). Health Hazards of Medical Waste and Its Disposal. Energy from Toxic Organic Waste for Heat and Power Generation.

[62] Makajic-Nikolic D., Petrovic N., Belic A., Rokvic M., Radakovic J.A., Tubic V. (2016). The Fault Tree Analysis of Infectious Medical Waste Management. J. Clean. Prod..

[63] Park H., Lee K., Kim M., Lee J., Seong S.-Y., Ko G. (2009). Detection and Hazard Assessment of Pathogenic Microorganisms in Medical Wastes. J. Environ. Sci. Health. Part A Toxic/Hazard. Subst. Environ. Eng..

[64] Blenkharn I., Odd C. (2008). Sharps Injuries in Healthcare Waste Handlers. Ann. Occup. Hyg..

[65] Udofia E.A., Gulis G., Fobil J. (2017). Solid Medical Waste: A Cross Sectional Study of Household Disposal Practices and Reported Harm in Southern Ghana. BMC Public Health.

[66] Jahangiri M., Rostamabadi A., Hoboubi N., Tadayon N., Soleimani A. (2016). Needle Stick Injuries and Their Related Safety Measures among Nurses in a University Hospital, Shiraz, Iran. Saf. Health Work.

[67] Gupta D.K., Singh M., Agarwal V.K., Sharma S., Mishra S. (2020). A Study of Contaminated Sharp Injury and Associated Morbidity among Health Care Workers. Int. J. Community Med. Public Health.

[68] Singh N., Tang Y., Ogunseitan O.A. (2020). Environmentally Sustainable Management of Used Personal Protective Equipment. Environ. Sci. Technol..

[69] Amsalu A., Worku M., Tadesse E., Shimelis T. (2016). The Exposure Rate to Hepatitis B and C Viruses among Medical Waste Handlers in Three Government Hospitals, Southern Ethiopia. Epidemiol. Health.

[70] Doddaiah V., Janakiram K., Javagal S. (2013). Seroprevalence of Hepatitis B Virus and Hepatitis C Virus in Healthcare Workers-Aims, Bg Nagara. Am. J. Life Sci..

[71] Shiferaw Y., Abebe T., Mihret A. (2011). Hepatitis B Virus Infection among Medical Aste Handlers in Addis Ababa, Ethiopia. BMC Res. Notes.

[72] Akpieyi A., Tudor T.L., Dutra C. (2015). The Utilisation of Risk-Based Frameworks for Managing Healthcare Waste: A Case Study of the National Health Service in London. Saf. Sci..

[73] Anagaw B., Shiferaw Y., Anagaw B., Belyhun Y., Erku W., Biadgelegn F., Moges B., Alemu A., Moges F., Mulu A. (2012). Seroprevalence of Hepatitis B and C Viruses among Medical Waste Handlers at Gondar Town Health Institutions, Northwest Ethiopia. BMC Res. Notes.

[74] Alemayehu T., Worku A., Assefa N. (2016). Medical Waste Collectors in Eastern Ethiopia Are Exposed to High Sharp Injury and Blood and Body Fluids Contamination. Prev. Inf. Cntrl..

[75] He L., Lu Z., Huang J., Zhou Y., Huang J., Bi Y., Li J. (2016). An Integrated Intervention for Increasing Clinical Nurses’ Knowledge of Hiv/Aids-Related Occupational Safety. Int. J. Environ. Res. Public Health.

[76] Saadeh R., Khairallah K., Abozeid H., Al Rashdan L., Alfaqih M., Alkhatatbeh O. (2020). Needle Stick and Sharp Injuries among Healthcare Workers: A Retrospective Six-Year Study. Sultan Qaboos Univ. Med. J..

[77] Khraisat F.S., Juni M.H., Salmiah M.S., Abd Rahman A., Hamdan-Mansour A. (2015). Needle Stick Injuries Prevalence among Nurses in Jordanian Hospitals. Int. J. Public Health Clin. Sci..

[78] De Carli G., Abiteboul D., Puro V. (2014). The Importance of Implementing Safe Sharps Practices in the Laboratory Setting in Europe. Biochem. Med..

[79] Ghanei Gheshlagh R., Aslani M., Shabani F., Dalvand S., Parizad N. (2018). Prevalence of Needlestick and Sharps Injuries in the Healthcare Workers of Iranian Hospitals: An Updated Meta-Analysis. Environ. Health Prev. Med..

[80] Kakizaki M., Ikeda N., Ali M., Enkhtuya B., Tsolmon M., Shibuya K., Kuroiwa C. (2011). Needlestick and Sharps Injuries among Health Care Workers at Public Tertiary Hospitals in an Urban Community in Mongolia. BMC Res. Notes.

[81] Matsubara C., Sakisaka K., Sychareun V., Phensavanh A., Ali M. (2017). Prevalence and Risk Factors of Needle Stick and Sharp Injury among Tertiary Hospital Workers, Vientiane, Lao Pdr. J. Occup. Health.

[82] Doggalli D.N. (2014). Hazards and Public Health Impacts of Hospital Waste. J. Appl. Res..

[83] Shaaban H., Alghamdi H., Alhamed N., Alziadi A., Mostafa A. (2018). Environmental Contamination by Pharmaceutical Waste: Assessing Patterns of Disposing Unwanted Medications and Investigating the Factors Influencing Personal Disposal Choices. J. Pharmacol. Pharm. Res..

[84] Sasu S., Kümmerer K., Kranert M. (2011). Assessment of Pharmaceutical Waste Management at Selected Hospitals and Homes in Ghana. Waste Manag. Res..

[85] Kümmerer K. (2010). Pharmaceuticals in the Environment. Annu. Rev. Environ. Resour..

[86] Sangion A., Gramatica P. (2016). Hazard of Pharmaceuticals for Aquatic Environment: Prioritization by Structural Approaches and Prediction of Ecotoxicity. Environ. Int..

[87] Chi T., Zhang A., Zhang X., Li A.-D., Zhang H., Zhao Z. (2020). Characteristics of the Antibiotic Resistance Genes in the Soil of Medical Waste Disposal Sites. Sci. Total Environ..

[88] Forsberg K.J., Reyes A., Wang B., Selleck E.M., Sommer M.O.A., Dantas G. (2012). The Shared Antibiotic Resistome of Soil Bacteria and Human Pathogens. Science.

[89] Ghasemi L., Yousefzadeh S., Rastkari N., Naddafi K., Shariati Far N., Nabizadeh R. (2018). Evaluate the Types and Amount of Genotoxic Waste in Tehran University of Medical Science’s Hospitals. J. Environ. Health Sci. Eng..

[90] Connor T.H., Lawson C.C., Polovich M., McDiarmid M.A. (2014). Reproductive Health Risks Associated with Occupational Exposures to Antineoplastic Drugs in Health Care Settings: A Review of the Evidence. J. Occup. Environ. Med..

[91] Shahrasbi A.A., Afshar M., Shokraneh F., Monji F., Noroozi M., Ebrahimi-Khojin M., Madani S.F., Ahadi-Barzoki M., Rajabi M. (2014). Risks to Health Professionals from Hazardous Drugs in Iran: A Pilot Study of Understanding of Healthcare Team to Occupational Exposure to Cytotoxics. EXCLI J..

[92] Capoor M.R., Bhowmik K.T. (2017). Cytotoxic Drug Dispersal, Cytotoxic Safety, and Cytotoxic Waste Management: Practices and Proposed India-Specific Guidelines. Indian J. Med. Paediatr. Oncol. Off. J. Indian Soc. Med. Paediatr. Oncol..

[93] Simegn W., Dagnew B., Dagne H. (2020). Knowledge and Associated Factors Towards Cytotoxic Drug Handling among University of Gondar Comprehensive Specialized Hospital Health Professionals, Institutional-Based Cross-Sectional Study. Environ. Health Prev. Med..

[94] Viegas S., Ladeira C., Costa-Veiga A., Perelman J., Gajski G. (2017). Forgotten Public Health Impacts of Cancer—An Overview. Arch. Ind. Hyg. Toxicol..

[95] Kovács R., Csenki Z., Bakos K., Urbányi B., Horváth Á., Garaj-Vrhovac V., Gajski G., Gerić M., Negreira N., López de Alda M. (2015). Assessment of Toxicity and Genotoxicity of Low Doses of 5-Fluorouracil in Zebrafish (Danio Rerio) Two-Generation Study. Water Res..

[96] Borowy I. (2020). Medical Waste: The Dark Side of Healthcare. História Ciências Saúde-Manguinhos.

[97] Njagi N.A., Oloo M.A., Kithinji J., Kithinji M.J. (2012). Health-Care Waste Incineration and Related Dangers to Public Health: Case Study of the Two Teaching and Referral Hospitals in Kenya. J. Community Health.

[98] de Titto E., Savino A. (2019). Environmental and Health Risks Related to Waste Incineration. Waste Manag. Res..

[99] Jang Y.-C., Lee C., Yoon O.-S., Kim H. (2006). Medical Waste Management in Korea. J. Environ. Manag..

[100] Federico M., Pirani M., Rashid I., Caranci N., Cirilli C. (2010). Cancer Incidence in People with Residential Exposure to a Municipal Waste Incinerator: An Ecological Study in Modena (Italy), 1991–2005. Waste Manag..

[101] Santoro M., Minichilli F., Linzalone N., Coi A., Maurello M.T., Sallese D., Bianchi F. (2016). Adverse Reproductive Outcomes Associated with Exposure to a Municipal Solid Waste Incinerator. Ann. Dell’istituto Super. Sanita.

[102] Domingo J.L., Marquès M., Mari M., Schuhmacher M. (2020). Adverse Health Effects for Populations Living near Waste Incinerators with Special Attention to Hazardous Waste Incinerators. A Review of the Scientific Literature. Environ. Res..

[103] Saria J.A. (2016). Levels of Heavy Metals in Bottom Ash from Medical Waste Incinerators in Dar Es Salaam. J. Multidiscip. Eng. Sci. Stud..

[104] Ephraim P.I., Ita A., Eusebius I.O. (2013). Investigation of Soils Affected by Burnt Hospital Wastes in Nigeria Using Pixe. SpringerPlus.

[105] Dash A., Kumar S., Singh R.K. (2015). Thermolysis of Medical Waste (Waste Syringe) to Liquid Fuel Using Semi Batch Reactor. Waste Biomass Valoriz..

[106] Fang S., Jiang L., Li P., Bai J., Chang C. (2020). Study on Pyrolysis Products Characteristics of Medical Waste and Fractional Condensation of the Pyrolysis Oil. Energy.

[107] Ismail Z.Z., Talib A.R. (2016). Recycled Medical Cotton Industry Waste as a Source of Biogas Recovery. J. Clean. Prod..

[108] Olaifa A., Govender R.D., Ross A.J. (2018). Knowledge, Attitudes and Practices of Healthcare Workers About Healthcare Waste Management at a District Hospital in Kwazulu-Natal. S. Afr. Fam. Pract..

[109] Aung T.S., Luan S., Xu Q. (2019). Application of Multi-Criteria-Decision Approach for the Analysis of Medical Waste Management Systems in Myanmar. J. Clean. Prod..

[110] Woolridge A., Hoboy S., Letcher T.M., Vallero D.A. (2019). Chapter 27—Medical Waste. Waste.

[111] Elnour A.M., Moussa M.M.R., El-Borgy M.D., Fadelella N.E.E., Mahmoud A.H. (2015). Impacts of Health Education on Knowledge and Practice of Hospital Staff with Regard to Healthcare Waste Management at White Nile State Main Hospitals, Sudan. Int. J. Health Sci..

[112] Babanyara Y.Y., Aliyu A., Gana B.A., Musa M. (2020). A Review of the Knowledge, Attitude, and Practices of Healthcare Wastes Workers (Hcws) on Medical Waste in Developing Countries. Risks and Challenges of Hazardous Waste Management: Reviews and Case Studies.

